# Maternal hypothyroidism in mice influences glucose metabolism in adult offspring

**DOI:** 10.1007/s00125-020-05172-x

**Published:** 2020-05-30

**Authors:** Yasmine Kemkem, Daniela Nasteska, Anne de Bray, Paula Bargi-Souza, Rodrigo A. Peliciari-Garcia, Anne Guillou, Patrice Mollard, David J. Hodson, Marie Schaeffer

**Affiliations:** 1 Institute of Functional Genomics, CNRS, Inserm U1191, University of Montpellier, F-34094 Montpellier, France; 2 Institute of Metabolism and Systems Research, University of Birmingham, Edgbaston, UK; 3 COMPARE University of Birmingham and University of Nottingham, Midlands, Edgbaston, Nottingham, UK; 4 Centre for Endocrinology, Diabetes and Metabolism, Birmingham Health Partners, Birmingham, UK; 5 Department of Physiology and Biophysics, Institute of Biological Sciences, Federal University of Minas Gerais, Belo Horizonte, MG Brazil; 6 Morphophysiology and Pathology Sector, Department of Biological Sciences, Federal University of São Paulo, Diadema, SP Brazil

**Keywords:** Beta cell function, Calcium imaging, Diabetes, Hypothyroidism, Pancreas

## Abstract

**Aims/hypothesis:**

During pregnancy, maternal metabolic disease and hormonal imbalance may alter fetal beta cell development and/or proliferation, thus leading to an increased risk for developing type 2 diabetes in adulthood. Although thyroid hormones play an important role in fetal endocrine pancreas development, the impact of maternal hypothyroidism on glucose homeostasis in adult offspring remains poorly understood.

**Methods:**

We investigated this using a mouse model of hypothyroidism, induced by administration of an iodine-deficient diet supplemented with propylthiouracil during gestation.

**Results:**

Here, we show that, when fed normal chow, adult mice born to hypothyroid mothers were more glucose-tolerant due to beta cell hyperproliferation (two- to threefold increase in Ki67-positive beta cells) and increased insulin sensitivity. However, following 8 weeks of high-fat feeding, these offspring gained 20% more body weight, became profoundly hyperinsulinaemic (with a 50% increase in fasting insulin concentration), insulin-resistant and glucose-intolerant compared with controls from euthyroid mothers. Furthermore, altered glucose metabolism was maintained in a second generation of animals.

**Conclusions/interpretation:**

Therefore, gestational hypothyroidism induces long-term alterations in endocrine pancreas function, which may have implications for type 2 diabetes prevention in affected individuals.

**Electronic supplementary material:**

The online version of this article (10.1007/s00125-020-05172-x) contains peer-reviewed but unedited supplementary material, which is available to authorised users.

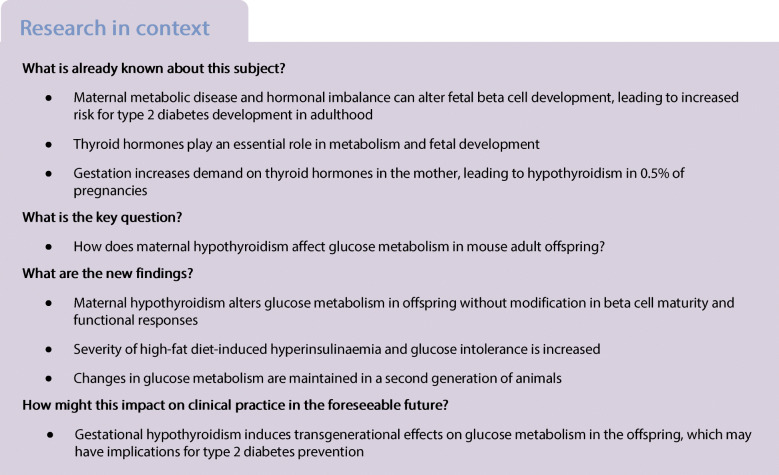



## Introduction

Type 2 diabetes and hypothyroidism are two major public health issues, affecting ~9% and 2%, respectively, of the population worldwide [1, 2]. These endocrine pathologies alter whole body metabolism and can sometimes be related, without presenting a common aetiology [3, 4]. Type 2 diabetes arises from a complex interplay between genetic and environmental factors [5]. In particular, the fetal environment plays a key role in the establishment of a functional beta cell mass [6]. Changes in the intrauterine milieu can modify beta cell differentiation and proliferation in the fetus, leading to long-term effects on glucose metabolism [7].

Different maternal conditions alter circulating concentrations of nutrients and hormones, which might impact beta cell development in utero. First, pre-existing metabolic disorders, such as malnutrition, obesity and diabetes, have been linked to increased susceptibility of the offspring to chronic diseases, such as hypertension and diabetes [7, 8]. In mice, maternal diabetes induces fetal hyperglycaemia and hyperinsulinaemia through accelerated endocrine pancreas development, predisposing to type 2 diabetes at later stages [9]. Second, gestation itself leads to important metabolic and hormonal modifications. For instance, gestational diabetes, occurring in 13% of pregnancies [10], alters endocrine pancreas maturation in the fetus and constitutes a risk factor for type 2 diabetes in adulthood [9]. In addition, gestation increases demand on thyroid hormones in the mother, leading to hypothyroidism in 0.5% of pregnancies [11].

As master metabolic gatekeepers, the thyroid hormones thyroxine (3,3′,5,5′ tetraiodothyronine; T_4_) and 3,3′,5-triiodothyronine (T_3_) play an essential role in metabolism and fetal development. Maternal hypothyroidism is associated with deficits in fetal growth and cardiac, nervous and bone maturation [12, 13]. Such dramatic effects result from a complete dependence of the fetus on maternal thyroid hormones until mid-gestation in mice and second trimester of pregnancy in humans [14, 15], and a continued influence of maternal thyroid hormones at later stages [14]. At the level of the pancreas, different studies have demonstrated important effects of thyroid hormones on beta cell development and maturation [16, 17]. These effects can be direct, through specific interactions with cognate receptors on beta cells [18], or indirect, through modification of the availability of growth factors [16], thereby altering glucose metabolism and insulin resistance [3]. Although a recent study showed that fetal hypothyroidism in sheep leads to increased beta cell proliferation and hyperinsulinaemia in the fetus [17], the consequences of gestational hypothyroidism on beta cell function in adult offspring remains unexplored. Thus, we sought to investigate the effects of gestational hypothyroidism on beta cell maturity and function, glucose metabolism, and susceptibility to metabolic stress such as high-fat diet (HFD) in adult mouse offspring and their descendants.

## Methods

### Mice

**Fig. 1:**
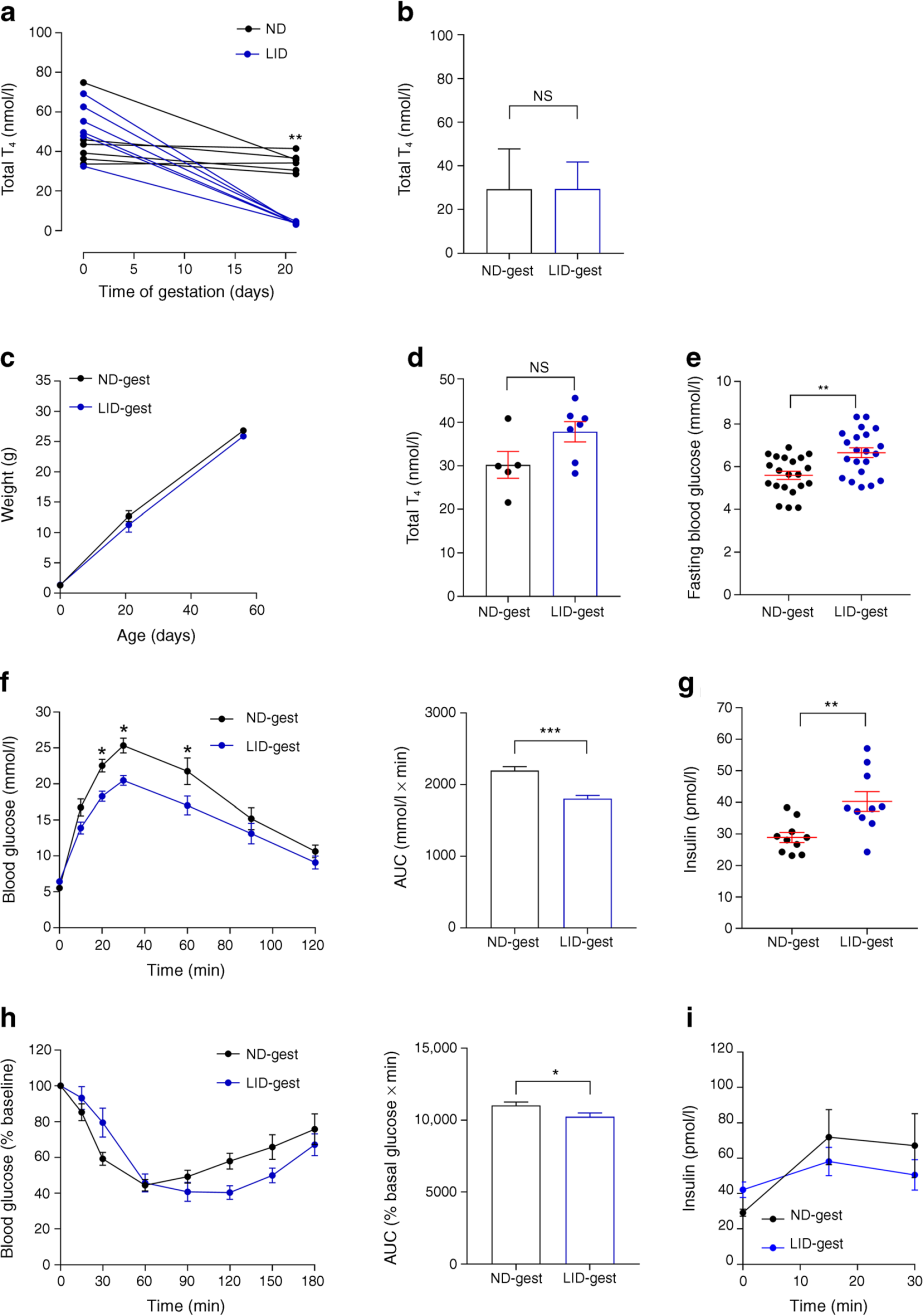
Congenital hypothyroidism alters glucose metabolism in male offspring. (**a**) Total plasma T_4_ concentration in dams before gestation and postpartum (*n* = 4–11 mice/group, Mann–Whitney *U* test). Statistical measurement is at 20 days for ND vs LID. (**b**) Measurement of total T_4_ in pooled blood from male and female newborn pups (at P0, pooled blood from at least four pups per measurement, 15–17 pups in total from at least two different dams in each groups, Mann–Whitney). (**c**–**i**) Adult male offspring (8–10 weeks of age) were analysed. (**c**) Male offspring weight over time after birth (*n* = 15 mice/group, one-way ANOVA). (**d**) Total plasma T_4_ concentration in adult male offspring (*n* = 5–7 mice/group, Mann–Whitney). (**e**) Fasting blood glucose in adult male offspring (*n* = 20 mice/group, Mann–Whitney). (**f**) IPGTT using 3 g glucose/kg body weight (*n* = 10 mice/group, two-way ANOVA) and AUC analysis (*n* = 10 mice/group, Mann–Whitney). (**g**) Fasting basal insulin concentrations (*n* = 10 mice/group, Mann–Whitney). (**h**) ITT (0.75 U insulin/kg) and AUC analysis (*n* = 10 mice/group, Mann–Whitney). (**i**) In vivo insulin responses to glucose (3 g/kg), (*n* = 9 mice/group, two-way ANOVA). *p < 0.05, **p < 0.01, ***p < 0.001 using the tests indicated above. Data are mean ± SEM. gest, gestation

Animal studies were conducted according to the European guidelines for animal welfare (2010/63/EU). Protocols were approved by the Institutional Animal Care and Use Committee (CEEA-LR-1434) and the French Ministry of Agriculture (APAFIS#13044). Mice were housed in a conventional facility on a 12 h light/12 h dark cycle and were given chow and water ad libitum. FVB/NJ mice were purchased from Janvier-SAS (Le Genest-St-Isle, France). Hypothyroidism in gestating mice was induced by feeding animals with an iodine-deficient diet (low-iodine diet, LID) supplemented with 0.15% propylthiouracil (PTU) (LID +0.15% PTU diet TD.95125, Envigo, USA) [19] from the first day post-coitus. Offspring were subsequently fed with normal diet (ND) until age 8–10 weeks and then fed with either ND or HFD (63% energy from fat) (Safe Diets, France) for 8 weeks. Analysed offspring were from at least three independent breeding pairs per group. Offspring numbers were evenly distributed between dams for analysis and allocated to treatment groups in a randomised manner to ensure that all states were represented in the different experiment arms. Second generation animals originated from two different male or three different female breeders born to two different hypothyroid mothers. The two males were each bred to three different females, and the three females each bred to one male, specifically purchased for this purpose. Experimenters were blind to group assignment during data analysis. No data were excluded unless animals died during experimentation. IPGTT, ITT and glucose-stimulated insulin secretion in vivo (GSIS) tests were as described [20, 21]. We chose a glucose dose of 3 g/kg body weight to ensure full beta cell challenge and generation of an insulin peak [22]. Mice were euthanised by decapitation after asphyxiation with CO_2_. Total trunk blood was collected and total T_4_ was measured in plasma in duplicate using a total T_4_ Elisa kit (EIA-1781, DRG International). T_4_ concentrations were measured in plasma (50 μl) isolated from blood collected at the tail for a limited number of dams, to ensure proper nursing of pups. To measure T_4_ concentrations in neonates, pooling of blood from a minimum of four animals at day 0 postpartum (P0) was necessary for each measurement. Litters from two to three different dams, corresponding to a total of 15 to 17 P0 pups were used. A flow chart of experimental design and mouse selection for the different assays in depicted in electronic supplementary material (ESM) Fig. 1.

### Confocal imaging and image analysis

Pancreas preparation and antibody labelling were as described [21]. Briefly, pancreases were fixed overnight in 4% paraformaldehyde and sliced (100 μm slices) on a vibratome (Leica, Germany) before immunostaining. Antibodies used were: rabbit anti-Ki67 (1:200, CliniSciences, France), guinea pig anti-insulin (1:400, Abcam, UK) and mouse anti-glucagon (1:200, Sigma, USA). Nuclei were labelled using DAPI (Sigma). Images (top 20 μm at the surface of slices) were acquired using a Zeiss (Germany) LSM 780 confocal microscope. Images were analysed using Imaris (Bitplane, UK), Volocity (Perkin Elmer, USA) and ImageJ (NIH, USA). For quantifications, four slices were randomly selected from at least three animals/group, and all islets present analysed. A priori, this is sufficiently-powered to detect a minimum 1.2-fold difference with an SD of 40%, a power of 0.9, and *α* = 0.05 (G*Power 3.1, Germany). The proportion of proliferative beta cells was obtained by dividing the number of Ki67^+^ nuclei by the total number of nuclei of insulin^+^ cells in islets, as described [21]. Beta cell mass was measured by dividing the area occupied by insulin^+^ cells by the total area of the tissue.

### Islet isolation and live calcium imaging of isolated islets

Islets were hand-picked after collagenase digestion of the whole pancreas [23], and cultured (5% CO_2_, 37°C) in RPMI medium containing 10% FCS, 100 units/ml penicillin, and 100 μg/ml streptomycin. Islets were loaded with Fluo-8 (AAT Bioquest, USA; Cat#21082) dissolved in DMSO containing 20% pluronic acid. Islets were then imaged using an X-Light spinning disk system (Crest, Italy) coupled to a Ti-E base (Nikon, Japan) and ×10/0.4/air objective (Nikon, Japan). Excitation was delivered at *λ* = 458–482 nm using a Spectra X-light engine (Lumencor, USA), with emitted signals detected at *λ* = 500–550 nm using a Delta Evolve EM-CCD (Teledyne Photometrics, USA). Imaging buffer contained (in mmol/l) 120 NaCl, 4.8 KCl, 24 NaHCO_3_, 0.5 Na_2_HPO_4_, 5 HEPES, 2.5 CaCl_2_, 1.2 MgCl_2_, and 3–17 d-glucose. Data were analysed using ImageJ, with traces presented as *F*/*F*_min_ where *F* = fluorescence at any timepoint and *F*_min_ = minimum fluorescence. No data were excluded unless the cells displayed a clear non-physiological state (i.e. impaired viability).

### Islet isolation and real-time quantitative RT-PCR

Pancreatic islets were hand-picked after collagenase digestion of whole pancreas, as described [23]. Total RNA from mouse islets was extracted using RNeasy microkit (Qiagen, Germany) following the manufacturer’s instructions. Reverse transcription was carried out using random hexamer oligonucleotides and SuperScriptIII Reverse Transcriptase (2000 U; Invitrogen, LifeTechnologies, USA). The reverse transcription product was diluted according to the efficiency curve and submitted in duplicates to real-time quantitative PCR using LightCycler 480 SYBR Green I Master (Roche, Switzerland) in 7500 System (Applied Biosystems, USA). Selection of housekeeping genes was performed using NormFinder software (Denmark, https://moma.dk/normfinder-software) [24]. PCR reactions were performed following the conditions: 95°C for 5 min, followed by 45 cycles of 95°C for 10 s and 72°C for 30 s, and the melting curve was performed from 65°C up to 97°C for 1 min. *C*_t_ values were recorded for each gene (listed in ESM Table 1) and normalised to the geometric mean of *Ppia* and *Mrpl32. C*_t_ values were then expressed relative to offspring from ND*-fed animals*. A list of primers used is shown in ESM Table 1.

### Statistical analysis

Values are represented as mean ± SEM. Statistical tests were performed using GraphPad Prism (USA). Normality was tested using D’Agostino–Pearson test, and comparisons were made using either unpaired Student’s *t* test, or two-tailed Mann–Whitney *U* test, as appropriate. Multiple comparisons were made using one-way or two-way ANOVA followed by Bonferroni’s post hoc test. A *p* value was considered significant at *p < 0.05, **p < 0.01, ***p < 0.001.

## Results

### Maternal hypothyroidism alters glucose homeostasis in adult offspring

Gestational hypothyroidism in female mice was induced from the first day post-coitus by administration of an LID supplemented with PTU, known to block thyroid hormone synthesis and conversion [19]. This approach led to severe hypothyroidism, shown by a marked postpartum decrease in total T_4_ hormone concentrations (Fig. 1a), as previously described [19, 25, 26]. Since the model is well established, confirmation of T_4_ hormone concentration, which requires large amounts of plasma, was limited to a reduced number of animals to avoid stressing dams and ensure proper nursing of pups. Analysis of total T_4_ concentrations in pooled blood from newborn pups showed that concentrations were comparable between animals born to euthyroid and hypothyroid mothers, suggesting normal thyroid function at birth in both groups (Fig. 1b). Although male mice born to hypothyroid mothers did not display significant changes in weight gain compared with control mice (Fig. 1c) and were euthyroid (Fig. 1d), glucose metabolism in adults (8–10 weeks) was increased. In particular, male offspring of iodine-deficient mothers presented higher fasting blood glucose concentration (Fig. 1e) but improved glucose tolerance (Fig. 1f). Insulin sensitivity was altered (Fig. 1h), fasting insulin concentrations were increased (Fig. 1g) and glucose-stimulated insulin release during IPGTT was unchanged (Fig. 1i).

**Fig. 2:**
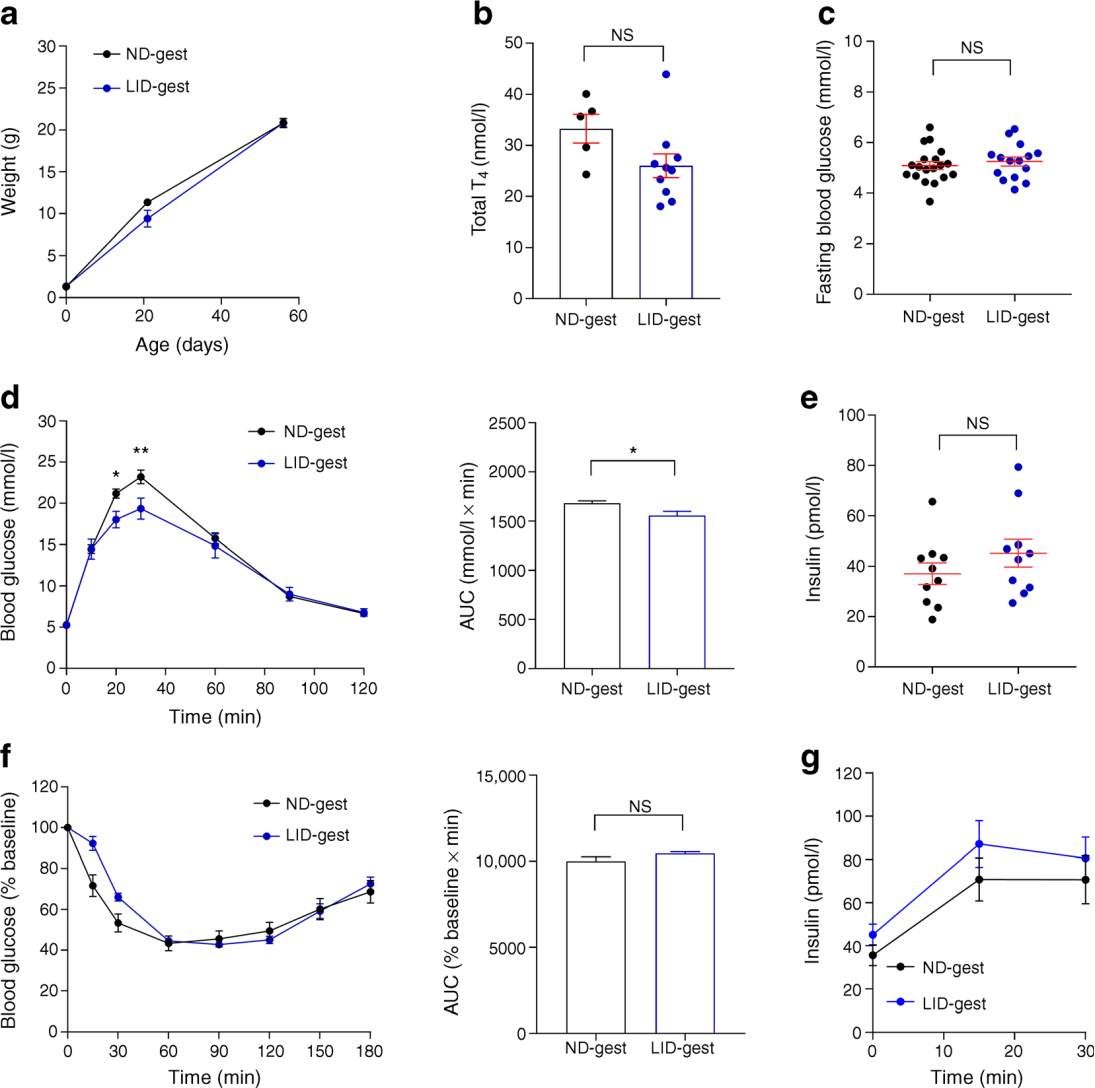
Congenital hypothyroidism alters glucose metabolism in female offspring. Adult female offspring (8–10 weeks of age) were analysed. (**a**) Female offspring weight over time after birth (*n* = 15 mice/group, one-way ANOVA). (**b**) Total plasma T_4_ concentration in adult female offspring (*n* = 5–10 mice/group, Mann–Whitney *U* test). (**c**) Fasting blood glucose in adult female offspring (*n* = 15 mice/group, Mann–Whitney). (**d**) IPGTT (3 g/kg) (*n* = 10 mice/group, two-way ANOVA) and AUC analysis (*n* = 10 mice/group, Mann–Whitney). (**e**) Fasting insulin concentrations (*n* = 10 mice/group, Mann–Whitney). (**f**) ITT (0.75 U/kg) and AUC analysis (*n* = 10 mice/group, Mann–Whitney). (**g**) In vivo insulin responses to glucose (3 g/kg) (*n* = 10 mice/group, one-way ANOVA). *p < 0.05, **p < 0.01 using the tests indicated above. Data are mean ± SEM. gest, gestation

In female offspring (8–10 weeks of age), alterations in glucose metabolism were also observed, albeit less pronounced (Fig. 2). Female mice born to hypothyroid mothers presented similar weight gain from birth to adulthood compared with control mice (Fig. 2a), and were euthyroid (Fig. 2b). Although fasting blood glucose concentrations were similar to those in control mice (Fig. 2c), female offspring of iodine-deficient mothers displayed improved glucose tolerance (Fig. 2d). However, insulin sensitivity (Fig. 2f), fasting insulin concentration (Fig. 2e) and glucose-stimulated insulin release (Fig. 2g) remained unchanged.

### Gestational hypothyroidism alters beta cell proliferation

**Fig. 3:**
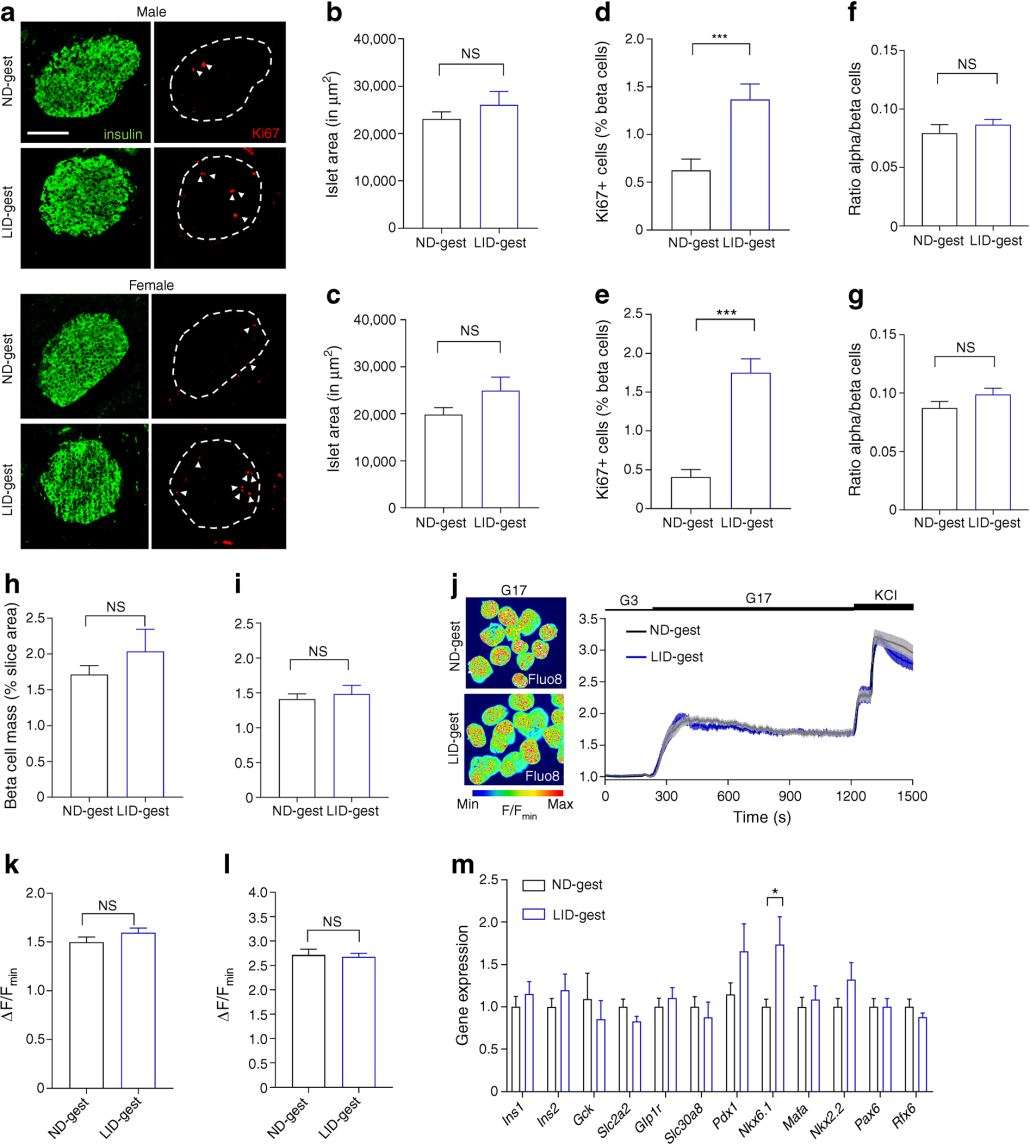
Gestational hypothyroidism alters beta cell proliferation without changes in calcium fluxes and beta cell identity. Adult offspring (8–10 weeks of age) were analysed. (**a**) Representative confocal images of pancreatic islets in male and female offspring (scale bar, 100 μm, 5 μm Z-projection; red, Ki67; green, insulin). Dashed circles delineate islets and arrowheads indicate Ki67^+^ beta cell nuclei. (**b**, **c**) Quantification of islet area in male (**b**) and female (**c**) offspring (*n* = 5 males and 10 females/group, Mann–Whitney *U* test). (**d**, **e**) Quantification of beta cell proliferation in male (**d**) and female (**e**) offspring (measured as % of beta cells positive for Ki67) (*n* = 5 males and 10 females/group, Mann–Whitney). (**f**, **g**) Quantification of alpha cell/beta cell ratio in male (**f**) and female (**g**) offspring (*n* = 5 males and 10 females/group, Mann–Whitney). (**h**, **i**) Beta cell mass, measured as ratio of insulin^+^ staining area by total slice area, in adult males (**h**) and females (**i**) (*n* = 3 mice/group, Mann–Whitney). (**j**–**l**) Representative images, with mean traces (right) and summary bar graphs (**k**, **l**) showing no changes in the amplitude of 17 mmol/l glucose- (**k**) and glucose + KCl- (**l**) stimulated Ca^2+^ rises in LID-gest male offspring (*n* = 44–61 islets/9–12 mice, Mann–Whitney). Note that the mean values in (**j**) do not correspond exactly to those in (**k**), since the time to Ca^2+^ peak following glucose stimulation was offset by 90 s in a number of islets. (**m**) Expression profiles of key beta cell markers in islets from male offspring, measured by RT-qPCR. Data were normalised by the geometric mean of *Ppia* and *Mrpl32. C*_t_ values are expressed as fold increase relative to offspring from *ND-*fed control. Data are presented as mean ± SEM (*n* = 10 mice/group, Mann–Whitney). *p < 0.05, ***p < 0.001 using the tests indicated above. Data are mean ± SEM. gest, gestation

Morphometric analyses of pancreatic sections showed that islet area was similar in offspring born to hypothyroid and euthyroid mothers (Fig. 3a–g). However, Ki67 labelling in beta cells, a marker of proliferation, was consistently found to be ~twofold higher both in males and females born to hypothyroid mothers (Fig. 3a, d, e), without changes in islet size, alpha to beta cell ratio or beta cell mass (Fig. 3b, c, f–i). Frequency distribution of islet sizes did not show a clear shift in islet sizes, since only a small increase in the percentage of very large islets in animals born to hypothyroid mothers could be evidenced (ESM Fig. 2).

Suggesting that defects in insulin secretion are distal to the triggering pathway, glucose- and KCl-stimulated Ca^2+^ rises were unchanged in islets from male animals born to hypothyroid mothers (Fig. 3j–l). Furthermore, the abundance of selected genes responsible for maintenance of beta cell identity and maturity remained unchanged (*Mafa*, *Pdx1*, *Rfx6*, *Pax6*, *Ins1*, *Ins2*, *Slc2a2*, *Gck and Glp1r*), with the exception of increased *Nkx6.1* mRNA expression levels (Fig. 3m).

### Gestational hypothyroidism renders offspring more susceptible to metabolic stress

**Fig. 4:**
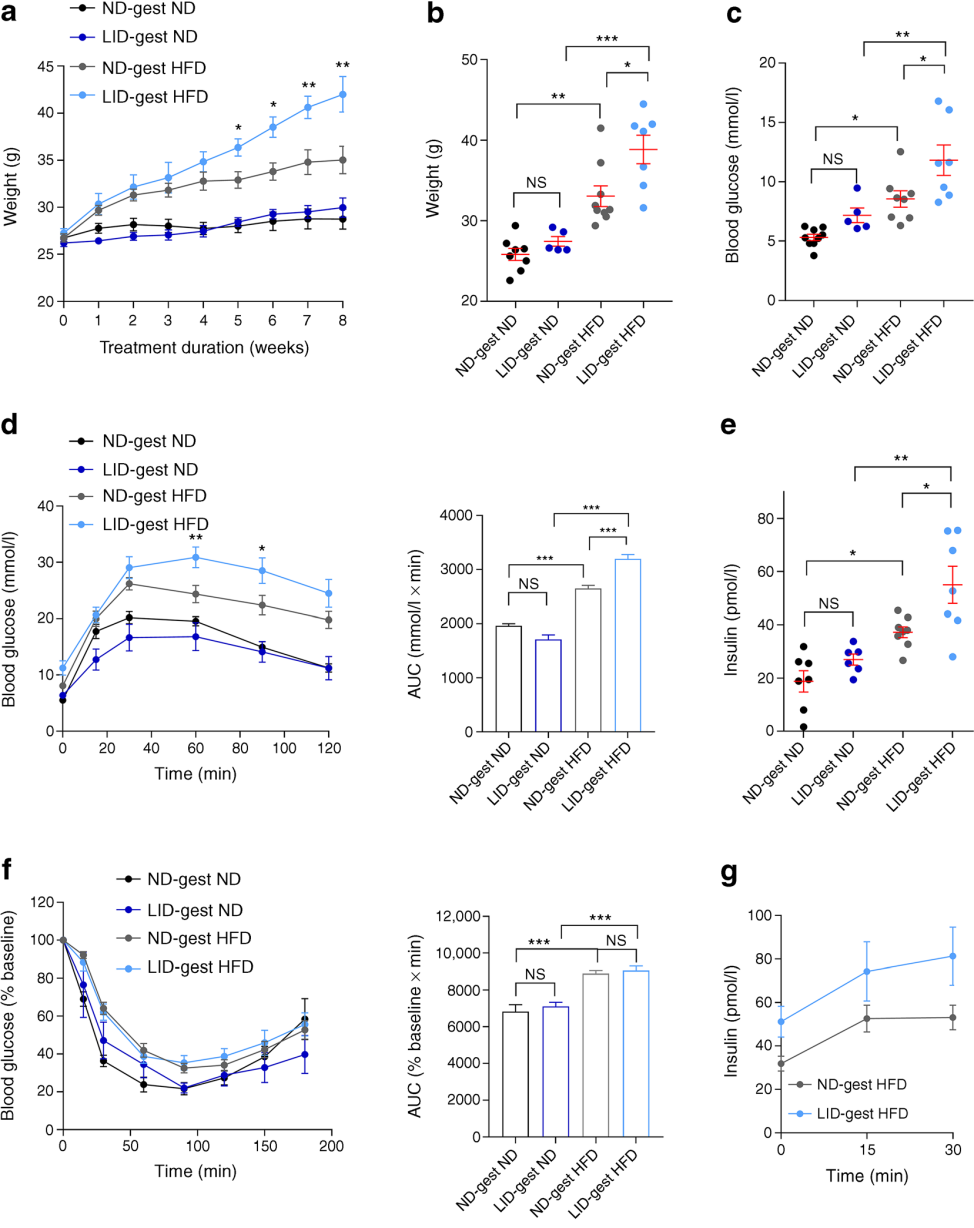
Gestational hypothyroidism renders offspring more susceptible to metabolic stress. Adult male offspring (16–18 weeks of age) were analysed. (**a**) Growth curve post initiation of feeding treatments showing increased weight gain in male offspring from hypothyroid mothers (*n* = 5–8 mice/group, two-way ANOVA; asterisks denote differences between LID-gest HFD and ND-gest HFD groups). (**b**) Animal weight at the end of feeding treatment (8 weeks) (*n* = 5–8 mice/group, one-way ANOVA). (**c**) Fasting blood glucose at the end of feeding treatment (8 weeks) (*n* = 5–8 mice/group, one-way ANOVA). (**d**) IPGTT (3 g/kg) (*n* = 5–8 mice/group, two-way ANOVA) and AUC analysis (*n* = 5–8 mice/group, one-way ANOVA). (**e**) Fasting basal insulin concentrations (*n* = 5–8 mice/group, one-way ANOVA). (**f**) ITT (0.75 U/kg) and AUC analysis (*n* = 5–8 mice/group, one-way ANOVA). (**g**) In vivo insulin responses to glucose (3 g/kg), (*n* = 8 mice/group, one-way ANOVA). *p < 0.05, **p < 0.01, ***p < 0.001 using the tests indicated above. Data are mean ± SEM. ND-gest ND, ND during gestation then ND; LID-gest ND, LID during gestation then ND; ND-gest HFD, ND during gestation then HFD; LID-gest HFD, LID during gestation then HFD

**Fig. 5:**
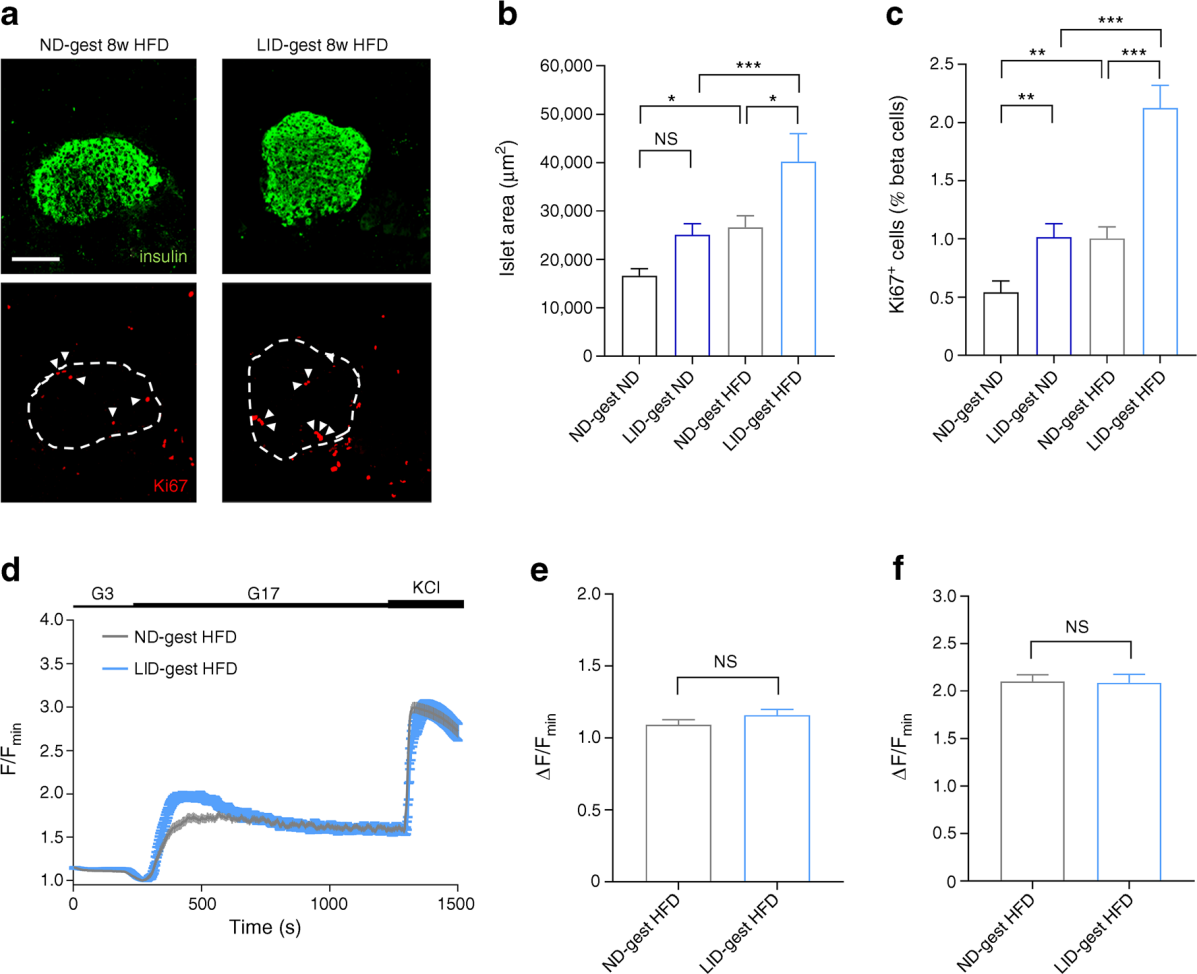
Metabolic stress-induced changes in beta cell proliferation and calcium fluxes are comparable between offspring from hypothyroid and euthyroid mothers. Adult male offspring (16–18 weeks of age) were analysed. (**a**) Representative confocal images of pancreatic islets in male offspring (scale bar, 100 μm, 5 μm Z-projection; red, Ki67; green, insulin). Dashed circles delineate islets and arrowheads indicate Ki67^+^ beta cell nuclei. (**b**) Quantification of islet area (*n* = 5–8 mice/group, one-way ANOVA). (**c**) Quantification of beta cell proliferation (measured as % of beta cells positive for Ki67) (*n* = 5–8 mice/group, one-way ANOVA). (**d**–**f**) Mean traces (**d**) and summary bar graphs (**e**, **f**) showing no changes in the amplitude of 17 mmol/l glucose- (**e**) and glucose +10 mmol/l KCl- (**f**) stimulated Ca^2+^ rises in LID-gest offspring fed HFD (*n* = 58–76 islets/6–7 mice/group, Mann–Whitney *U* test). *p < 0.05, **p < 0.01, ***p < 0.001 using the tests indicated above. Data are mean ± SEM. ND-gest ND, ND during gestation then ND; LID-gest ND, LID during gestation then ND; ND-gest HFD, ND during gestation then HFD; LID-gest HFD, LID during gestation then HFD

We next explored whether gestational hypothyroidism would influence compensatory responses to metabolic stress in adult male offspring. Compared with controls, animals born to hypothyroid mothers displayed increased weight gain following HFD feeding (Fig. 4a, b). However, weight gain was comparable when the same animals were fed ND for an equivalent duration (Fig. 4a, b). While HFD increased fasting glucose (Fig. 4c), and induced glucose intolerance (Fig. 4d) in all animals examined, the defect was most severe in offspring born to hypothyroid mothers, despite similar insulin sensitivity to age-matched controls (Fig. 4f). An analogous effect was seen in fasting insulin concentrations (Fig. 4e), with pronounced basal hyperinsulinaemia present in offspring born to hypothyroid mothers, despite the absence of any changes in insulin concentrations following glucose challenge (Fig. 4g). Interestingly, although maternal hypothyroidism altered glucose homeostasis in young adult offspring, differences in insulin sensitivity and glucose intolerance were not significant in older animals (16–18 weeks of age) kept on standard chow (Fig. 4). We note however that seven animals were required to reach statistical significance for female animals, so the lack of effect in male animals might reflect a sample size issue. At the morphological level, HFD increased islet size and beta cell proliferation to a similar extent in offspring from both hypothyroid and euthyroid mothers (Fig. 5a–c), as expected [27]. No differences in glucose- or KCl-stimulated Ca^2+^ rises were detected between the two groups of animals (Fig. 5d–f), suggesting that altered ionic fluxes were unlikely to contribute to the impaired glucose tolerance observed in HFD-fed offspring from hypothyroid mothers.

### The effects of gestational hypothyroidism on glucose homeostasis are transgenerational

**Fig. 6:**
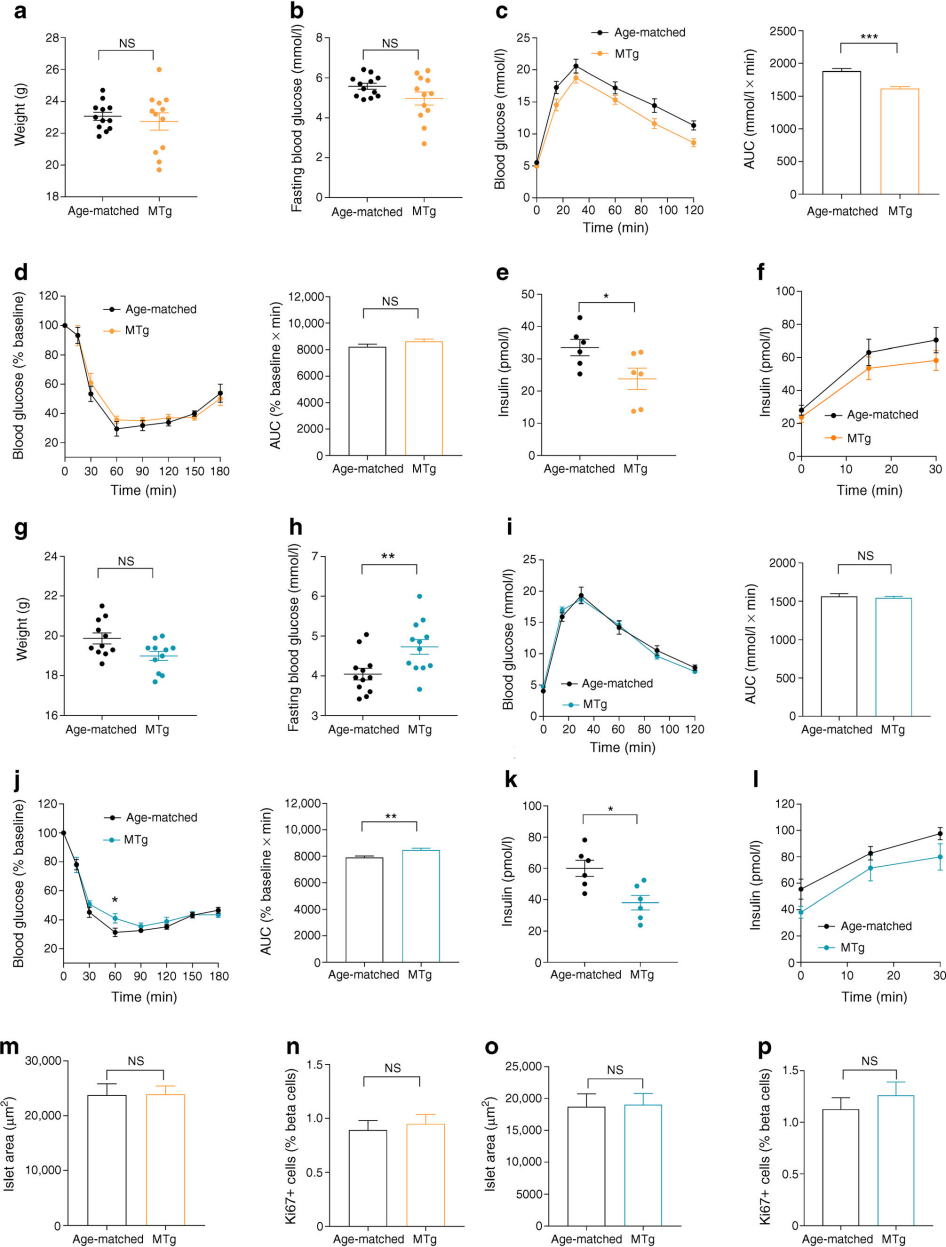
Effects of gestational hypothyroidism on glucose homeostasis in a second generation of animals. Adult MTg mice (8–10 weeks of age) were compared with age-matched controls. (**a**) Male weight at adult age (8–10 weeks) (*n* = 12 mice/group, Mann–Whitney *U* test). (**b**) Fasting blood glucose in males (*n* = 12 mice/group, Mann–Whitney). (**c**) IPGTT in males (3 g/kg) and AUC analysis (*n* = 12 mice/group, Mann–Whitney). (**d**) ITT in males (0.75 U/kg) and AUC analysis (*n* = 12 mice/group, Mann–Whitney). (**e**) Fasting insulin concentrations in males (*n* = 6 mice/group, Mann–Whitney). (**f**) In vivo insulin responses to glucose in males (3 g/kg), (*n* = 6 mice/group). (**g**) Female weight at adult age (8–10 weeks) (*n* = 12 mice/group, Mann–Whitney). (**h**) Fasting blood glucose in females (*n* = 12 mice/group, Mann–Whitney). (**i**) IPGTT in females (3 g/kg) and AUC analysis (*n* = 12 mice/group, Mann–Whitney). (**j**) ITT in females (0.75 U/kg) and AUC analysis (*n* = 12 mice/group, two-way ANOVA [left] and Mann–Whitney [right]). (**k**) Fasting insulin concentrations in females (*n* = 6 mice/group, Mann–Whitney). (**l**) In vivo insulin responses to glucose in females (3 g/kg), (*n* = 6 mice/group). (**m**) Quantification of islet area in males (*n* = 6 mice/group, Mann–Whitney). (**n**) Quantification of beta cell proliferation in males (*n* = 6 mice/group, Mann–Whitney). (**o**) Quantification of islet area in females (*n* = 6 mice/group, Mann–Whitney). (**p**) Quantification of beta cell proliferation in females (*n* = 6 mice/group, Mann–Whitney). *p < 0.05, **p < 0.01, ***p < 0.001 using the tests indicated above. Data are mean ± SEM. MTg, second generation of male (in orange) and female (in cyan) offspring from males born to hypothyroid mothers

We finally investigated whether alterations in glucose metabolism persisted in a second generation of animals. To do this, glucose tolerance, insulin resistance, fasting insulin concentrations and beta cell proliferation were all assessed in adult offspring (8–10 weeks of age) originating from male mice born to hypothyroid mothers. While the second generation of males presented similar weight and fasting blood glucose compared with control age-matched animals (Fig. 6a, b), glucose tolerance remained improved (Fig. 6c). This change was despite normal insulin sensitivity (Fig. 6d), decreased fasting insulin concentration (Fig. 6e) and normal insulin secretion in response to intraperitoneal glucose (Fig. 6f). Second generation female offspring also presented similar weight compared with control aged-matched animals (Fig. 6g). By contrast to males, glucose tolerance was unchanged (Fig. 6i), despite increased fasting blood glucose (Fig. 6h) and decreased insulin sensitivity (Fig. 6j), decreased fasting insulin concentration (Fig. 6k) and normal glucose-stimulated insulin release Fig. 6l). Finally, islet size and beta cell proliferation were unchanged in both male and female offspring (Fig. 6m–p). Comparable results were obtained in adult offspring (8–10 weeks of age) originating from female mice born to hypothyroid mothers (ESM Fig. 3). Thus, gestational hypothyroidism induces transgenerational changes in metabolism in both paternal and maternal lines, with differential effects in male and female offspring.

## Discussion

Circulating factors in utero can influence fetal endocrine pancreas development and lead to life-long alterations in glucose metabolism. Since gestation modulates thyroid hormone concentrations, which are known to play an important role in beta cell development and maturation [12, 17], we sought to investigate whether maternal hypothyroidism influences glucose homeostasis in adult offspring. We found that gestational hypothyroidism increased beta cell proliferation, altered glucose metabolism and increased the severity of HFD-induced obesity in offspring, without altering beta cell maturity and functional responses. Furthermore, alterations in glucose metabolism were maintained in a second generation of adults. These results therefore indicate that maternal hypothyroidism may exert transgenerational effects on glucose homeostasis, although primary effects on germ cells during the previous pregnancy cannot be completely excluded.

Hypothyroidism is one of the most common endocrine diseases during pregnancy and is mainly linked to dietary iodine deficiency, especially in low–middle income countries [3]. Thus, iodine deficiency in the diet constitutes a robust model to induce congenital hypothyroidism through decreases in circulating total T_4_ concentration during gestation [26]. While definitive confirmation of hypothyroidism would also require measurement of thyroid-stimulating hormone (TSH), commercial assay kits do not work reliably in mice. Exposure to hypothyroidism in utero has been reported to influence growth in other rodents [28, 29]. However, we could not detect significant differences in body mass between mice born to euthyroid or hypothyroid mothers. While this may reflect the model used, we note that intrauterine growth restriction does not necessarily correlate with altered body weights in neonates [30]. Indeed, in sheep, hypothyroidism in utero induced pancreatic beta cell proliferation and hyperinsulinaemia in the fetus [17], which would be expected to maintain growth rate.

Although the effects of thyroid hormone deficiency on fetal pancreas development were not assessed here, congenital hypothyroidism in mice altered glucose metabolism and stimulated beta cell proliferation in both adult male and female mouse offspring. This suggests impaired development of the fetal or postnatal pancreas, leading to long-term changes in glucose metabolism [31]. However, whether these long-term changes persist for the lifespan of the animal remains to be investigated. Interestingly, beta cell proliferation was not accompanied by an increase in islet size or beta cell mass or a clear shift in islet size range. This may be due to timing of analysis or relative sensitivies of beta cell proliferation vs islet size measures. In addition, we cannot exclude the possibility that proliferation is balanced by apoptosis or cell cycle progression block after mitosis, maintaining beta cell mass. This, however, remains to be investigated. Overall, the alterations were more pronounced in male offspring, possibly reflecting known sex-dependent effects of fetal hypothyroidism on glucose metabolism [32] and pointing to the importance of including both sexes in studies of this type. In fact, T_4_-dependent liver function and metabolism is sex-dependent, and sex is an important modifier of the extent of phenotypic manifestations of hypo- or hyperthyroidism, not only at the metabolic level, but also of functional, biochemical and molecular traits [33].

In line with our results, congenital hypothyroidism has been previously shown to induce long-term alterations in glucose metabolism in adult male rat offspring [31, 34]. In particular, insulin secretion was found to be decreased, in contrast to the lack of difference detected in the present study. The reasons for this are unknown, but may include the dynamic evolution of glucose metabolism with age (young adult vs mature animals), the proliferative status of beta cells (not assessed in rat studies), and/or species-related differences.

Since glucose-stimulated Ca^2+^ fluxes are a major triggering signal for insulin release [35], we hypothesised that long-term alterations in glucose metabolism induced by congenital hypothyroidism may be linked to changes in beta cell stimulus–secretion coupling. Previous studies on isolated islets from male rat offspring showed that maternal hypothyroidism led to impaired insulin secretion through a combination of different mechanisms, including alteration in glycolytic pathways and ATP sensitive K^+^ (K_ATP_) and L-type Ca^2+^ channel conductance [34]. However, both glucose- and KCl-induced Ca^2+^ rises were found to be unchanged in islets from male animals born to hypothyroid mothers. Thus, the changes in in vivo insulin responses and glucose metabolism described in the present study are likely to result from mechanisms distal to Ca^2+^ fluxes, such as amplyfying pathway (e.g. cAMP) or granule exocytosis. Alternatively, since thyroid hormones are crucial regulators of growth, development and metabolism in virtually all tissues, in particular during fetal stages [12], metabolic alterations may rise from a combination of modifications in different organs. For instance, congenital hypothyroidism has been shown to alter liver development [36], and modify glucose transporter expression, impairing glucose sensing in glucose-sensitive organs, including the liver and metabolic regions of the brain [37]. Further studies will be needed to explore both these possibilities.

In adults, beta cell proliferation is triggered in response to increased metabolic demand such as gestation and HFD feeding [27, 38]. Although triiodothyronine stimulates proliferation of rat beta cell lines [39], whether thyroid hormones contribute to beta cell proliferation in response to demand remains unclear. During fetal development, the prepartum surge in thyroid hormone is thought to induce a switch from beta cell proliferation to functional maturation [16, 40], thus explaining the maintenance of beta cell proliferation in islets of hypothyroid sheep fetuses [17]. It is likely that similar mechanisms are at play in offspring of hypothyroid mothers, although we cannot exclude an increase in beta cell proliferation due to increased metabolic demand and/or insulin resistance. The source of such increased demand is, however, unclear, especially since ND-fed offspring displayed increase insulin sensitivity. Since T_3_/T_4_ are pre-requisite for cell maturation [41], and because in vivo insulin responses to glucose were decreased, we analysed overall gene expression of key markers defining adult beta cell functional identity [42]. However, we could not detect major changes in adult offspring from hypothyroid mothers, in line with the Ca^2+^ imaging data, suggesting that beta cell de-differentiation/or lack of maturation is not a feature here.

In addition to altered glucose metabolism and increased beta cell proliferation, maternal hypothyroidism increased susceptibility to HFD-induced metabolic stress in adult male offspring. Although glucose tolerance was not measured by dosing glucose according to lean body mass, differences are likely to persist, given the profound effect on all metabolic variables. This result fits with previous data showing that environmental alterations during endocrine pancreas development can induce long-term consequences for glucose metabolism [7]. The results here support the notion that maternal hypothyroidism may increase risk of type 2 diabetes development in later life. This increased susceptibility may be linked to exacerbated HFD-induced hyperinsulinaemia, which has previously been shown to drive insulin resistance and diet-induced obesity [43]. Again, changes were independent of Ca^2+^ channel activity, suggesting that the insulin secretory defect may lie distal to the triggering pathway. Although female offspring were not analysed, similar results would be expected, since congenital hypothyroidism also affected glucose metabolism in female offspring. Suggesting the presence of normal thyroid function in neonates born to hypothyroid mothers, total T_4_ concentrations were similar to control animals born to euthyroid mothers. Thus, effects of reduced maternal T_4_ on pancreas development might be either indirect, through altered placental size or function, for instance, or direct during early development, while the fetus is entirely dependent on maternal T_4_ hormone. This, however, remains to be investigated. Notably, maternal hyperglycaemia has also been shown to result in altered glucose metabolism in the progeny and increased predisposition to diabetes [44]. Since hypothyroidism affects glucose metabolism and insulin resistance [45], the extent to which alterations in maternal glucose homeostasis might account for some of the findings remains to be deciphered.

Finally, we saw that altered glucose metabolism persisted in a second generation of offspring, albeit to a lesser extent, suggesting the presence of epigenetic changes. Such changes are likely to be imprinted as a result of thyroid hormone deprivation during fetal development, since epigenetic reprogramming occurs during gametogenesis and early embryogenesis [46], before being transmitted to the next generation. It has indeed been shown in other models of nutritional insult in pregnant mice (e.g. high-fat, methyl-deficient or low-protein diets) that induction of epigenetic marks in beta cells leads to altered function and diabetes risk later in life [47, 48]. These epigenetic changes might be in part mediated through changes in maternal thyroid hormone secretion, since nutritional status is an important regulator of thyroid activity [49]. In addition, since both liver and pancreas are affected by similar signalling pathways during development and both organs display remarkable plasticity following insult in adults, epigenetic markers are likely to affect other organs than the endocrine pancreas [50]. We concede, however, that identification of these epigenetic markers is needed, and that a multitude of other mechanisms at central and peripheral levels may also be involved in altered glucose homeostasis following changes in thyroid hormone concentrations. A multi-organ analysis is warranted to achieve a full understanding of the effects.

In summary, we show that gestational hypothyroidism induces transgenerational effects on glucose metabolism in the offspring, which may affect predisposition to type 2 diabetes development in response to metabolic stress.

## Electronic supplementary material

ESM (PDF 559 kb)

## Data Availability

The datasets generated during and/or analysed during the current study are available from the corresponding author on reasonable request.

## Abbreviations

HFD: High-fat diet
LID: Low-iodine diet
ND: Normal diet
PTU: Propylthiouracil
