# Supplementary material for: Maternal hypothyroidism in mice influences glucose metabolism in adult offspring
Source: Diabetologia. 2020 May 30;63(9):1822–35. doi: 10.1007/s00125-020-05172-x (PMC7406527; doi:10.1007/s00125-020-05172-x)
Supplement: Supplementary file 1 — (PDF 559 kb) [file 125_2020_5172_MOESM1_ESM.pdf]

ELECTRONIC SUPPLEMENTARY MATERIAL (ESM)

ESM TABLE 1. List of primers used for quantitative RT-PCR

**Symbol** GenBank#	**Primers**	**Sequences (5'- 3')**	**Amplicon length (bp)**
Mrpl32 NM_029271.2	Forward Reverse	AGGTGCTGGGAGCTGCTACA AAAGCGACTCCAGCTCTGCT	51
Ppia NM_008907.1	Forward Reverse	CAAACACAAACGGTTCCCAG TTCACCTTCCCAAAGACCAC	85
Ins1 NM_008386.4	Forward Reverse	CTTCAGACCTTGGCGTTGGA TGCTGGTGCAGCACTGATCC	67
Ins2 NM_001185083.2	Forward Reverse	CATCAGCAAGCAGGAAG CCCAGAGGAAGAGCAG	83
Gck NM_010292.5	Forward Reverse	CTTCACCTTCTCCTTCCCTG ATCTCAAAGTCCCCTCTCCT	150
Pax6 NM_001244198.2	Forward Reverse	TGAGAAGTGTGGGAACCAGC AAGTCTTCTGCCTGTGAGCC	70
Nkx2.2 NM_001077632	Forward Reverse	GCCTCCAATACTCCCTGC GGTCTCCTTGTCATTGTCCG	110
Slc2a2 NM_031197.2	Forward Reverse	CCCTGTTCCTAACCGGGATG TCCAGGCGAATTTATCCAGCA	87
Nkx6.1 NM_144955.2	Forward Reverse	CGAACAAACGAAGTACTTGGC TTTCTCCACTTGGTCCTGC	114
Mafa NM_194350.1	Forward Reverse	GAAGTGCCAGCTCCAGAG CGCCAACTTCTCGTATTTCTCC	100
Glp1r NM_021332.2	Forward Reverse	CCCATGGGGGATTGTCAAGT AGAAAGTTGACGCCGATAGCA	120
Slc30a8 NM_172816.4	Forward Reverse	CGCCTTTTGTATCCTGATTACC GTTGTAGCCAAAGTTCCGTTG	120
Pdx1 NM_008814	Forward Reverse	CCCTTTCCCGTGGATGAAATC GAATTCCTTCTCCAGCTCCAG	145
Rfx6 NM_001159389.1	Forward Reverse	TGCTTACCTGCTGGCTGAAA TGCATTTCTGATTTAACTCCCACC	96

ESM Figure 1. Flow chart of experimental design and mouse selection for the different assays.

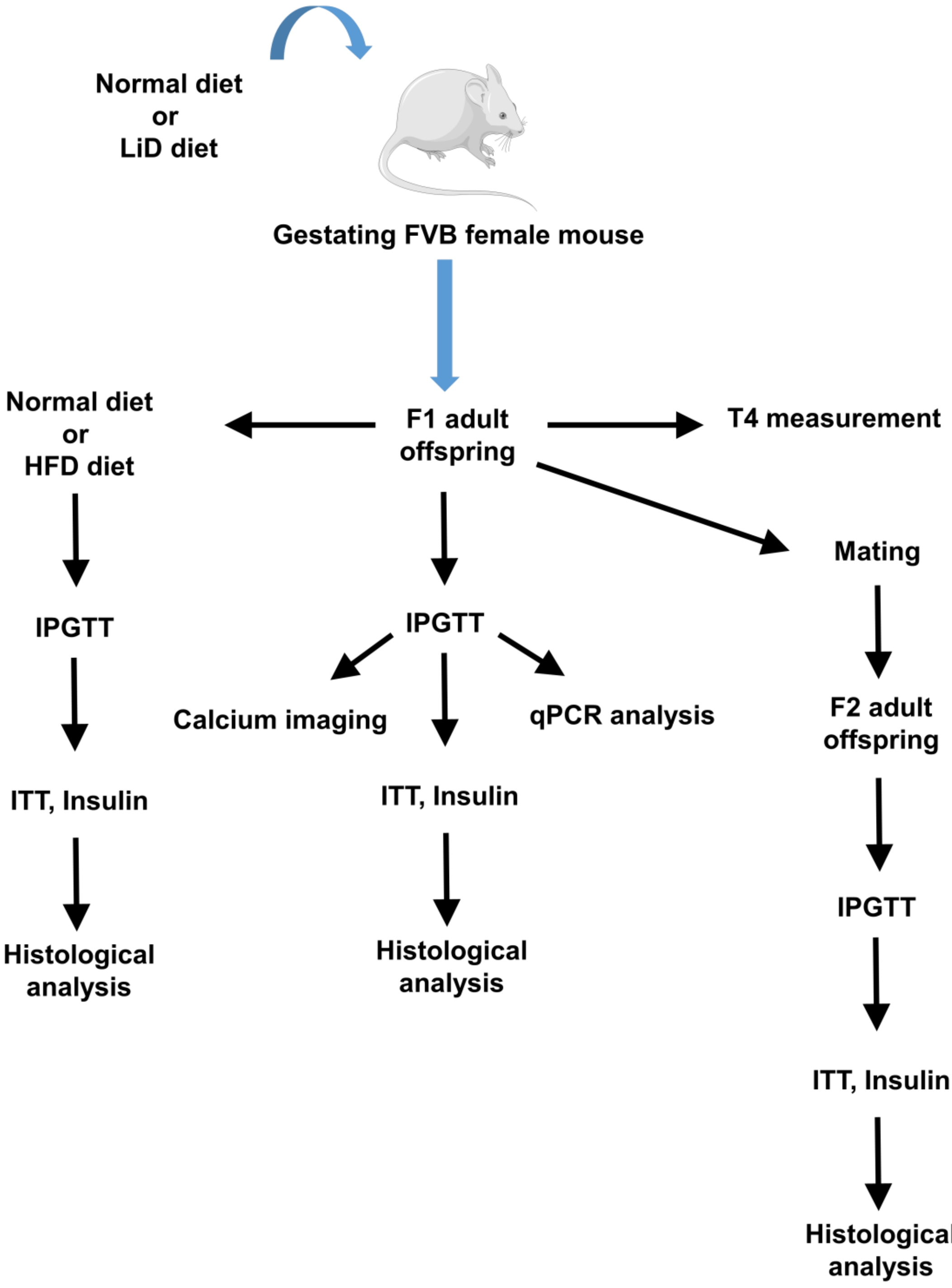

ESM Figure 2. Frequency distribution of islet sizes in the different groups. Frequency distribution of islet sizes (μm^2) as percent of total islets imaged in males (a) and females (b) (n = 5 males and 10 females/group).

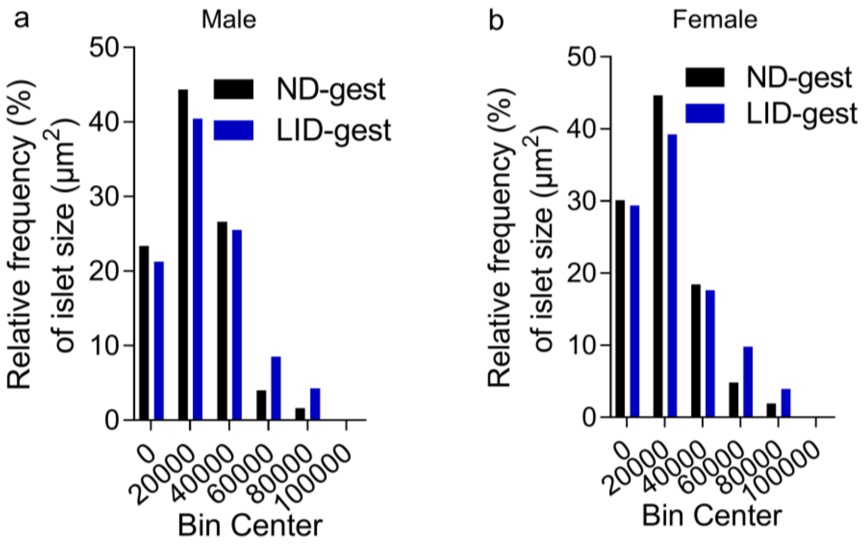

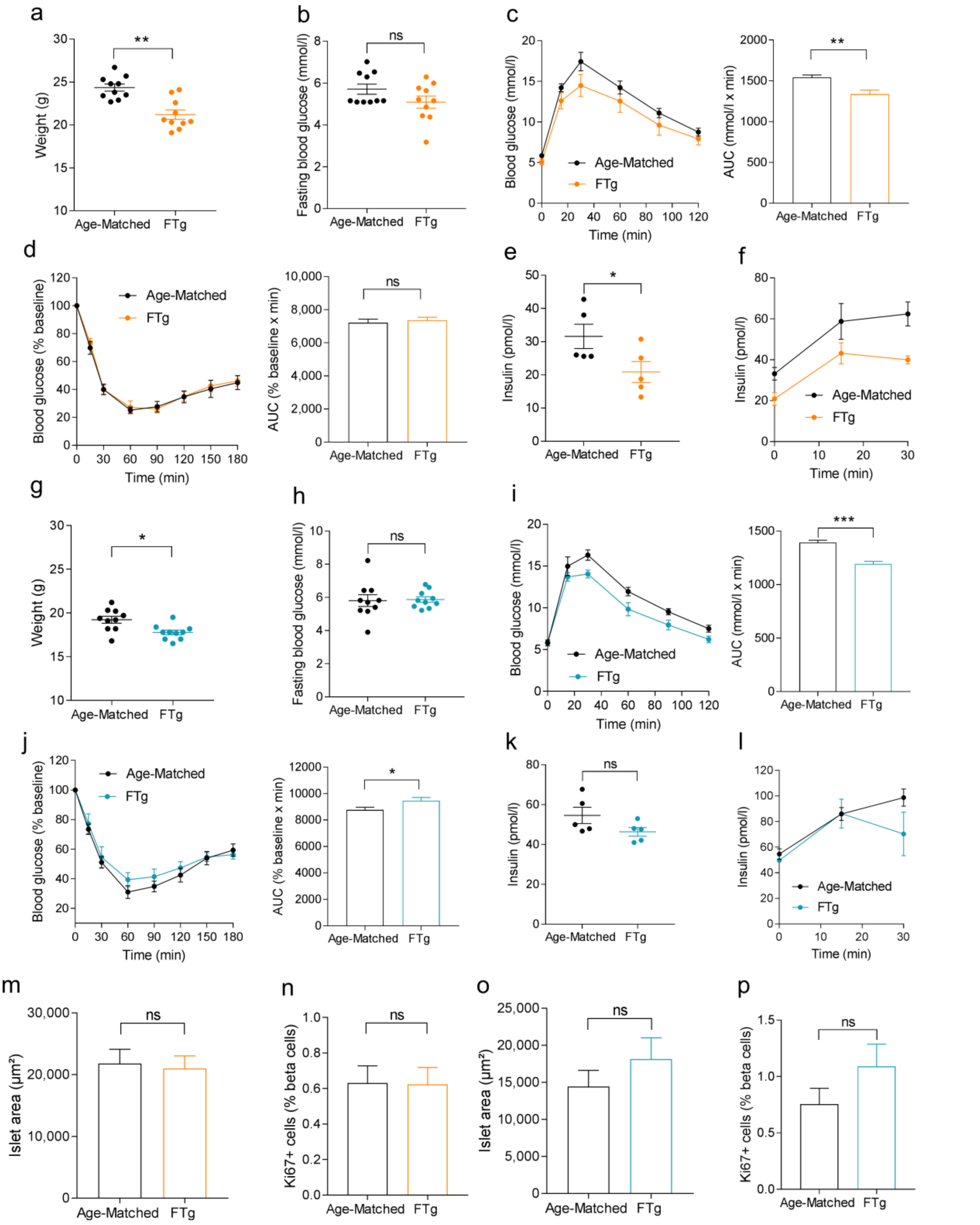

ESM Figure 3. Effects of gestational hypothyroidism on glucose homeostasis in a second generation of animals. FTg: second generation of male (in orange) and female (in cyan) offspring from females born to hypothyroid mothers. Adult FTg (8-10 weeks of age) were compared to age-matched controls (in black). Statistical notation: *p<0.05, **p<0.01,

***p<0.001. a) Male weight at adult age (8-10 weeks) (n = 10 mice/group, mean ± SEM, Mann-Whitney). b) Fasting blood glucose in males (n = 10 mice/group, mean ± SEM, Mann-Whitney). c) Glucose tolerance test in males (3 g/kg) and area under the curve (AUC) analysis (n = 10 mice/group, mean ± SEM, Mann-Whitney). d) Insulin tolerance test in males (0.75 U/kg) and AUC analysis (n = 10 mice/group, mean ± SEM, Mann-Whitney). e) Fasting insulin concentrations in males (n = 5 mice/group, mean ± SEM, Mann-Whitney). f) *In* vivo insulin responses to glucose in males (3 g/kg), (n = 5 mice/group, mean ± SEM). g) Female weight at adult age (8-10 weeks) (n = 10 mice/group, mean ± SEM, Mann-Whitney). h) Fasting blood glucose in females (n = 10 mice/group, mean ± SEM, Mann-Whitney). i) Glucose tolerance test in females (3 g/kg) and area under the curve (AUC) analysis (n = 10 mice/group, mean ± SEM, Mann-Whitney). j) Insulin tolerance test in females (0.75 U/kg) and AUC analysis (n = 10 mice/group, mean ± SEM, Two-way ANOVA (left panel) and Mann-Whitney (right panel)). k) Fasting insulin concentrations in females (n = 5 mice/group, mean ± SEM, Mann-Whitney). l) *In* vivo insulin responses to glucose in females (3 g/kg), (n = 6 mice/group, mean ± SEM). m) Quantification of islet area in males (n = 5 mice/group, mean ± SEM, Mann-Whitney). n) Quantification of beta cell proliferation in males (n = 5 mice/group, mean ± SEM, Mann-Whitney). o) Quantification of islet area in females (n = 5 mice/group, mean ± SEM, Mann-Whitney). p) Quantification of beta cell proliferation in females (n = 5 mice/group, mean ± SEM, Mann-Whitney).
